# Coronal Shear Fractures of the Distal Humerus

**DOI:** 10.3390/jfmk7010007

**Published:** 2022-01-06

**Authors:** Enrico Bellato, Riccardo Giai Via, Daniel Bachman, Ilaria Zorzolo, Antonio Marmotti, Filippo Castoldi

**Affiliations:** 1 Department of Surgical Sciences, San Luigi Gonzaga Hospital, University of Turin, 10124 Turin, Italy; filippo.castoldi@unito.it; 2 University of Turin, 10124 Turin, Italy; riccardogiaivia@gmail.com (R.G.V.); ilaria.zorzolo@gmail.com (I.Z.); 3 University of Missouri, Kansas City, MO 64110, USA; drbach01@gmail.com; 4 San Luigi Gonzaga Hospital, Orbassano, 10093 Turin, Italy; antonio.marmotti@inwind.it

**Keywords:** coronal fractures, shear fractures, capitellum, trochlea, distal humerus

## Abstract

Coronal shear fractures of the distal humerus are rare, frequently comminuted, and are without consensus for treatment. The aim of this paper is to review the current concepts on the diagnosis, classification, treatment options, surgical approaches, and complications of capitellar and trochlear fractures. Computed Tomography (CT) scans, along with the Dubberley classification, are extremely helpful in the decision-making process. Most of the fractures necessitate open reduction and internal fixation, although elbow arthroplasty is an option for comminuted fractures in the elderly low-demand patient. Stiffness is the most common complication after fixation, although reoperation is infrequent.

## 1. Introduction

Coronal shear fractures are partial or complete articular fractures of the distal humerus without involvement of the columns, which can affect the capitellum, the trochlea, or both.

**Figure 1:**
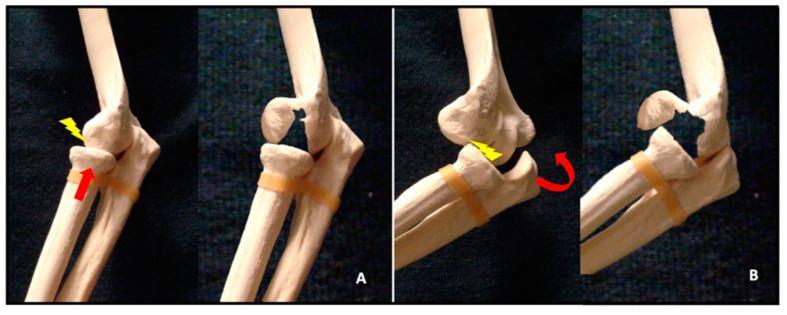
Pathogenic mechanisms of coronal shear fractures. The fracture is a result of: (**A**) an impact along the radius in full extension; or (**B**) posterolateral dislocation, often with posterior capitellar impaction; the radial head shears off the capitellum during the relocation of the elbow. The red arrow represents the direction of the force of the trauma. The yellow bolt points the site where the force is applied on the distal humerus.

Isolated fractures of the capitellum account for 1% of all elbow fractures and between 3% and 6% of distal humeral fractures [1,2,3,4]. Isolated fractures of the trochlea are even less common [5]. More than 80% of shear fractures occur in the female population and this has been attributed to increased cubitus valgus, recurvatum, and osteoporosis [6,7,8]. Shear fractures of the distal humerus can occur with a concomitant injury to the radial head and/or the lateral ulnar collateral ligament (LUCL) in up to 60% of patients [9]. Capitellar shear fractures commonly occur after low energy trauma. Two proposed mechanisms of injury relate to the two associated injuries. The first fracture mechanism is an indirect impact along the radius in full extension, which results in a vertical shear stress on the distal humerus caused by the radial head. The second is a posterolateral transient dislocation followed by spontaneous reduction, with the radial head shearing off the capitellum during the relocation of the elbow [10] (Figure 1).

These two potential mechanisms of injury could also explain the high incidence of associated injuries. A radial head fracture can occur both after a direct impaction onto the distal humerus and after an elbow posterolateral dislocation/subluxation. LUCL tears and coronoid fractures are typically subsequent to a posterolateral dislocation or subluxation [11,12].

These fractures are often challenging, as they can be characterized by small articular fragments, limited subchondral bone, bone impaction, and involvement of the medial epicondyle. In addition, when the posterolateral aspect of the distal humerus is involved, these fractures are at risk of avascular necrosis and nonunion [13,14]. This portion of the humerus is supplied by posterior perforating terminal vessels arising from the radial recurrent, radial collateral, and interosseous recurrent arteries [15].

## 2. Classification

Several classification systems for coronal shear fractures of the distal humerus have been described in the literature.

The Bryan–Morrey classification [16] is strictly focused on the capitellum. Type 1 (Hahn–Steinthal fracture) is a fracture that involves only the capitellum with attached subchondral bone. Type 2 (Kocher–Lorenz fracture) is an isolated fracture of the capitellum with the fragment consisting mainly on articular cartilage. Type 3 (Broberg–Morrey fracture) is an isolated capitellar fracture with comminution. A type 4 lesion (McKee fracture) [17] was further added and is a capitellar fracture that extends medially into the trochlea. Watts et al., reviewed 79 coronal shear fractures and observed that type 1 fracture was the most common one (47%) followed by type 4 (41%); type 2 occurred in 8% and type 3 in 4% [18].

**Figure 2:**
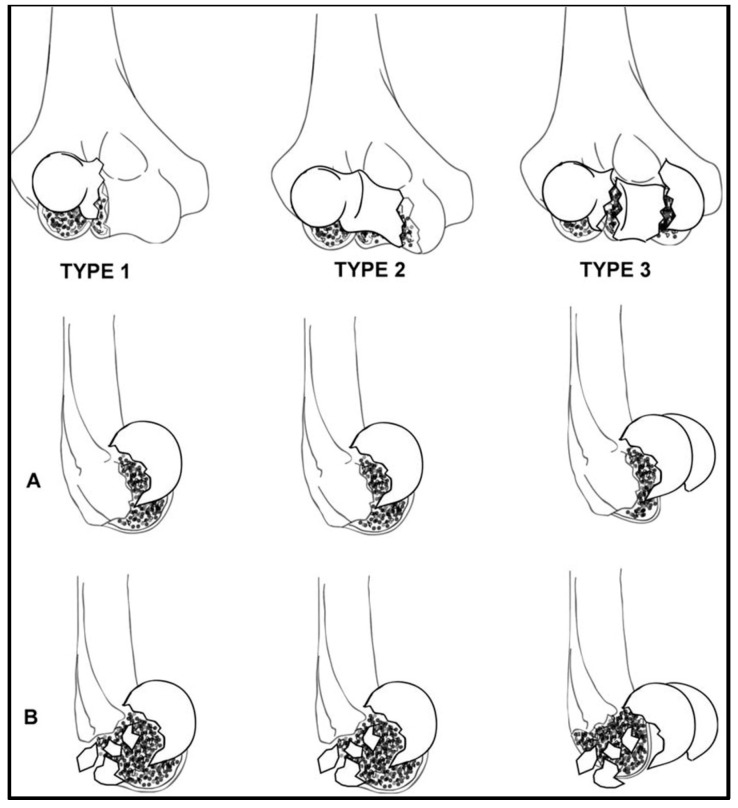
The Dubberley classification of capitellar and trochlear fractures. Subtype (**A**) fractures are not characterized by posterior comminution, while Subtype (**B**) show posterior comminution (From Dubberley J.H., Faber K.J. and Macdermid J.C. et al. Outcome after open reduction and internal fixation of capitellar and trochlear fractures. J Bone Joint Surg Am 2006; 88: 47 [6]; with permission).

Dubberley et al., published a more recent classification to better characterize capitellar fractures in order to drive the surgical management and to potentially predict the outcomes [6]. Type 1 is an isolated fracture of the capitellum extended to the most lateral portion of the trochlea (i.e., the equivalent to the Hahn–Steinthal fracture). Type 2 is a fracture of the capitellum and trochlea in a single fragment (i.e., the equivalent to the McKee fracture). Type 3 involves both the capitellum and the trochlea as separate fragments. These three types are further divided into subtype A—without posterior comminution or subtype B—with posterior comminution. Watson et al. proposed a rare “Type 4 fracture” which is a type 3 fracture with associated sagittal and axial plane fracture lines, creating multiple smaller fragments of the capitellum and trochlea [19]. This type of fracture is mainly seen in osteoporotic patients (Figure 2). One limitation of the Dubberley classification is that it does not include the associated injuries. There are no robust scientific data to draw conclusions on how these injuries influence both the treatment and the clinical outcomes.

The rare isolated fractures of the trochlea (T) can be included in the Dubberley classification as type 1T, differentiating it from fractures of the capitellum.

Ring and Jupiter proposed another descriptive classification [20]. Type 1 is a coronal shear fracture composed of a singular articular fragment that includes the capitellum and the lateral portion of the trochlea. Type 2 involves the lateral epicondyle. Type 3 is characterized by a further impaction of the metaphyseal bone behind the capitellum in the distal and posterior aspect of the lateral column. Type 4 is a fracture that includes the posterior aspect of the trochlea. Lastly, type 5 is a fracture that involves the medial epicondyle.

Finally, there is the AO-ASIF classification [21]. Coronal shear fractures are classified as type 13B3 (distal humerus, partial articular, frontal/coronal plane): 13B3.1 for capitellar fractures; 13B3.2 for trochlea fractures, and 13B3.3 for both capitellar and trochlea fractures.

## 3. Imaging

**Figure 3:**
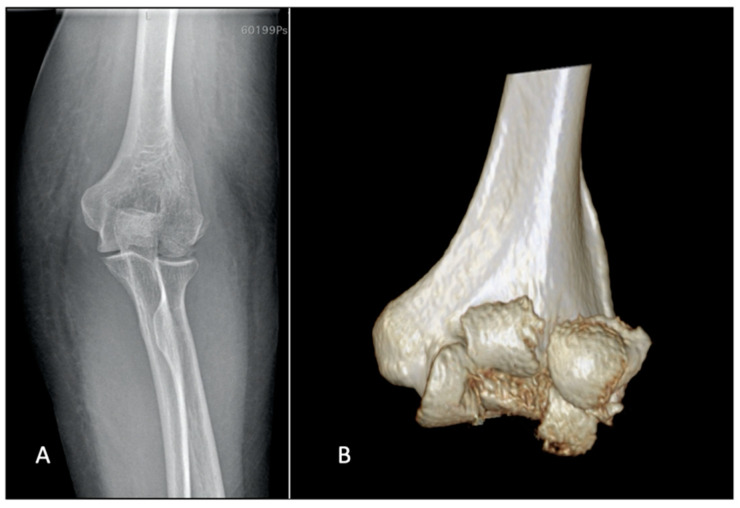
(**A**) The anteroposterior view barely shows involvement of the joint surface. (**B**) The CT scan portrays fracture of both the capitellum and the trochlea.

Plain X-rays represent the first step and are helpful in making the diagnosis. Anteroposterior views can be misleading because of the flexed position of the elbow and the flexed position of the fragments; sometimes the AP view can suggest comminution when additional sagittal plane fracture lines are observed (Figure 3).

**Figure 4:**
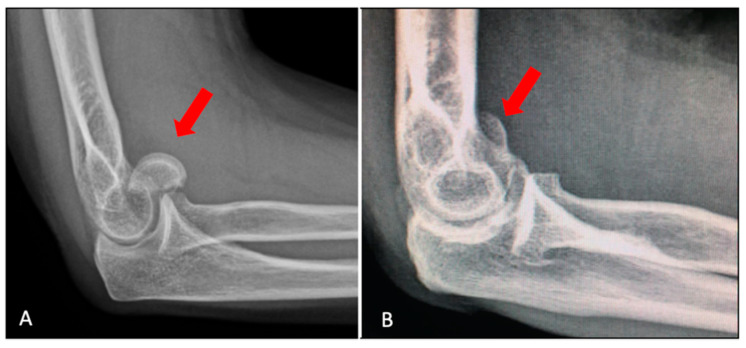
(**A**) Lateral X-ray showing the fracture fragment (red arrow) dislocated anterior to the distal humerus. (**B**) In cases of Bryan–Morrey Type II fracture, the injury looks like a loose body (red arrow).

The lateral view typically shows the fragment dislocated anterior to the distal humerus (Figure 4A), but shear fractures of mostly cartilage, i.e., the Byran–Morrey type II, in the lateral view, can look like a loose body (Figure 4B).

**Figure 5:**
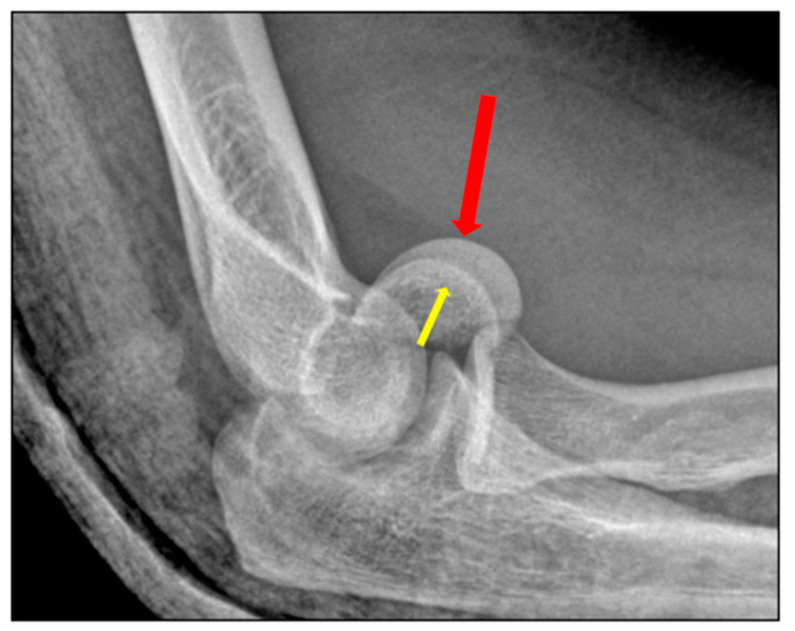
The “double arc sign” suggests the involvement of the capitellum (larger arc, red arrow) as well as the trochlea (smaller arc, yellow arrow).

A radiographic fat pad sign along with mechanism and physical examination consistent with elbow fracture should be highly suspicious [22]. In the lateral view, the “double arch sign” suggests involvement of both the capitellum (larger arc) and the trochlea (smaller arc) [17] (Figure 5).

In more complex fractures a ‘triple arc sign’ can be observed, which is suggestive of comminution, extensive involvement of the medial trochlea, or both [19].

**Figure 6:**
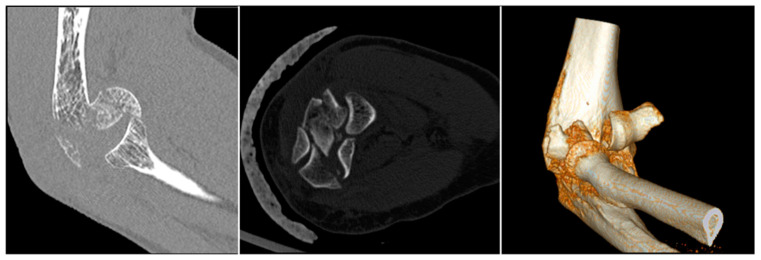
CT scan with 3D reconstruction helps the surgeon detect posterior comminution, analyze the number of fracture fragments, and understand the medial extension of the fracture.

A computed tomography (CT) scan with 3D reconstructions is very helpful for assessment of these patients [23]. CT allows for the precise evaluation of the posterior capitellum and medial trochlea. Impaction of the lateral column or the posterior trochlea and fractures of the lateral or medial epicondyles are assessed with more increased intrarater and interrater reliability than in plain films alone (Figure 6) [24].

## 4. Treatment Options and Results

### 4.1. Planning

In order to best plan the treatment, careful evaluation of the fracture morphology and associated injuries with the CT scan should be done. Posterior comminution poses higher risk of avascular necrosis and biomechanical failure of fracture fixation [15,18,25]. Medial extension drives the decision regarding the choice of surgical approach between a lateral, extended lateral, combined lateral and medial, or olecranon osteotomy approaches [15,19,26]. Impacted fracture fragments can be difficult to detect intraoperatively; if not identified pre-operatively, anatomic fracture reduction may not be possible. The associated radial head and more rarely, coronoid fractures, are diagnosed with the CT scan. Data regarding treatment of radial head fractures in the setting of coronal shear fractures are lacking. Nevertheless, the radial head should be treated as a fracture-dislocation, and fixation versus replacement should be performed depending on the level of comminution.

Associated LUCL tears are more difficult to diagnose pre-operatively, and the diagnosis is typically intraoperative. We believe that LUCL tears are common. Additionally, during the extended lateral approach the LUCL often needs to be detached if it is not already torn. Thus, the LUCL is almost always repaired.

### 4.2. Nonoperative Treatment

Few studies have reported on closed treatment with marginal results [1,27,28,29,30,31,32]. Ochner et al. described a closed maneuver performed acutely after the injury in noncomminuted fractures [28]. After extension and supination, the elbow undergoes traction and varus stress followed by flexion and pronation. Then, the elbow is immobilized in a cast for around 3 weeks. More recently, Catbush et al. described seven adult patients fracture conservatively treated and no signs of fracture displacement, osteoarthritis, or avascular necrosis [32]. After an average follow-up of 41.6 months, patients showed 97% of their active elbow flexion, 83% of elbow extension, and full pronation–supination. However, the study only included Bryan–Morrey type 1 fractures, which represent only around 40% of all the shear fractures. 

Although no robust scientific data exists that can help the surgeon decide, nonsurgical treatment is not usually recommended, as it is associated with a high risk of stiffness, pain, and post-traumatic arthritis [1,27,28]. Patients unfit to have surgery could have consideration of this option [26,33,34].

### 4.3. Fragment Excision

**Figure 7:**
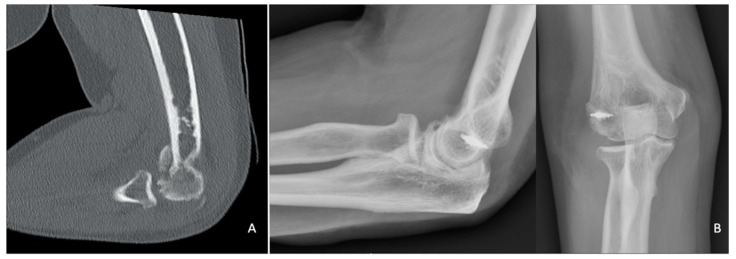
(**A**) Preoperative CT scan and (**B**) post-operative X-rays of a Bryan–Morrey Type II fracture treated with fragment excision and lateral collateral ligament repair using a suture anchor.

Excision is an option for small fracture fragments that are determined to be not fixable. The risk of intraarticular adhesions following the exposure of cancellous subchondral bone must be taken into consideration [35]. If the fracture involves the lateral trochlear ridge or further, excision is contraindicated, as it may destabilize the elbow [36]; however, there are no published data to support excision based on fracture classification [31]. Additionally, a medial collateral ligament tear contraindicates excision, due to the subsequent risk of chronic valgus instability [1,37] (Figure 7).

In the largest series describing excision, some including the trochlea, 29 patients were retrospectively reviewed (20 treated with excision and 9 with fixation), the results of excision were fair or poor with mean loss of ROM of 40° [1]. Fowles et al. tried an initial non-operative treatment and a delayed fragment excision if the elbow remained symptomatic. Their case series describing six patients reported worse outcomes in three patients treated after a mean of 5.7 months after trauma than the three treated within 24 h from trauma (91° vs. 141° of flexion, respectively—some residual pain in the group was later treated) [38].

In the rare cases of an isolated fracture of the trochlea, fragment excision is not a viable option because of the key role of this portion in elbow function [39].

### 4.4. Arthroscopic Treatment

Arthroscopy has been proposed both for fragment excision and fixation [40,41,42,43]. With theoretical benefits of minimal dissection, no dynamic stabilizers disrupted, and lower risk of infection, arthroscopy has the potential for a more precise articular fracture control, removal of loose bodies, and perhaps a better evaluation of the medial part of the joint [41,42,43,44]. However, it is highly technically demanding and neurovascular structures are at risk of iatrogenic injury.

Comminution can be a contraindication to arthroscopic fixation, especially if posterior, (i.e., Dubberley subtypes B) [45]. A relative contraindication is the Dubberley 3A fracture, as the capitellum and trochlea present as separate fragments [42,44].

Only few cases are reported in the literature and some lack intraoperative images. However, the early results appear promising. Van Nguyen, et al. [44], recently described their arthroscopic technique without clinical results. [44] First, varus stress is applied, followed by progressive elbow extension to reduce the fracture under arthroscopic visualization. Then, the reduction is refined using an arthroscopic periosteal elevator in 90° elbow flexion inserted through the anterolateral portal. A probe inserted through the posterolateral portal helps maintain the reduction, while the fragment is temporarily fixed with Kirschner wires. Definitive fixation is performed retrograde with percutaneous headless compression screws under the control of both fluoroscopy and arthroscopy.

### 4.5. Open Reduction and Internal Fixation (ORIF)

Most of the patients sustaining coronal shear fractures undergo ORIF. Due to the relatively uncommon nature of the pathology, the literature mostly consists of case series. Comparison studies regarding surgical approach and type of fixation are not reported. There are no data suggesting the optimal timing for surgery. However, earlier is likely best in order to reduce the amount of intra-articular scar tissue, subchondral bony resorption, and cartilaginous edema. Early surgery can potentially reduce the risk of bone avascular necrosis and accelerate the rehabilitation program.

### 4.6. Surgical Approaches

The ideal surgical approach depends on fracture characteristics, associated injuries, and surgeon preference.

Either a lateral, anterolateral, combined lateral and medial, or posterior superficial approach can be chosen, with the latter preferred when the fracture line extends far medially and posterior. Then, a deep lateral approach is usually sufficient. The Kocher interval is developed [46] and, if the lateral ulnar collateral ligament (LUCL) is intact, the capsule between the extensor carpi ulnaris and the anconeus should be incised as far anterior as possible, in order to preserve the LUCL integrity [47]. When the fracture line extends toward the trochlea, an extended Kocher approach might be preferred [48]— starting the incision at the supracondylar ridge and detaching the origins of the wrist extensors and brachioradialis from the lateral condyle. The origin of the LUCL sometimes needs to be detached as well to expose the posterior capitellum or mobilize impacted fragments in order to obtain an anatomic reduction and adequate fixation. Additionally, by raising the LUCL origin off the bone, the joint can be hinged open to allow for better exposure of the medial trochlea, while preserving the olecranon and medial soft tissues. In certain cases, the lateral epicondyle is fractured and the joint can be exposed by simple mobilization of the fragment, with the LUCL remaining attached to the bone [49]. Whenever the LUCL or the lateral epicondyle is detached, it needs a careful reinsertion, with either suture anchors or bone tunnel fixation, in order to avoid posterolateral rotatory instability.

An anterolateral approach has been described utilizing an “S” shaped incision: the brachioradialis and the radial nerve are retracted laterally and the biceps medially [50]. An anterior longitudinal incision through the brachialis and the capsule follows. This is a particularly good option for Dubberley type 2A and 3A fractures [51]. The disadvantages are that this approach is not extensile, it requires retraction of nerves, and it does not allow visualization of the posterior aspect of the capitellum.

When better visualization of the trochlea is needed, a separate medial “over the top” approach, described by Hotchkiss, that spares the anterior bundle of the medial collateral ligament, can be used [6,20,52].

In cases where severe medial and posterior comminution is observed, an olecranon osteotomy is an option. However, it is important to note, as this approach preserves both the medial and lateral ulnar collateral ligaments, the most anterior portion of the distal humerus is not clearly exposed even with extreme elbow flexion. A separate lateral window is necessary to do so, with detachment of the extensor carpi radialis longus, the brachialis, and the anterior capsule of the humerus. A triceps-sparing approach with subperiosteal elevation of collateral ligaments origin allows excellent visualization, but the drawback is the associated risk of iatrogenic instability. This approach is a good option to treat a severely comminuted fracture with an elbow replacement.

### 4.7. Fixation Techniques

#### 4.7.1. Isolated Screw Fixation

Screw fixation is the most frequently used type of fixation. Several types, including cortical, cancellous small fragment, Herbert, and headless compression screws, have been proposed [31].

**Figure 8:**
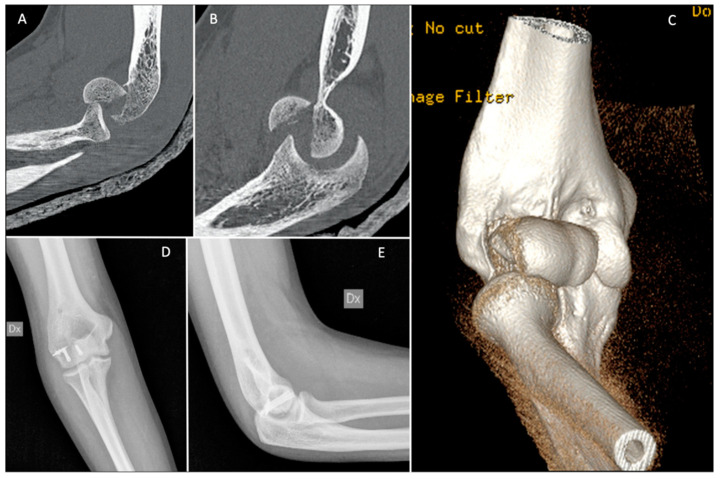
(**A**–**C**) Pre-operative CT scan; and (**D**,**E**) post-operative X-rays of a Dubberley 2A fracture fixed with independent antigrade headless screws.

Isolated screws are sufficient in some cases, including those of Dubberley subtype A fractures. After reducing the fracture by applying a clamp to obtain compression, the provisional fixation is obtained with Kirschner wires. These are usually the guide wires for the cannulated screws. If not, provisional Kirschner wires should be placed along the fracture margins so as not to interfere with placement of headless screws. We suggest the use of at least two screws and a divergent pattern (Figure 8) [53,54].

Screws with heads need to be countersunk within the articular surface, but we prefer headless variable pitch screws. Matache et al., have recently demonstrated that the proximal third of the lateral trochlear ridge, on average around 5 mm in width, is a non-articulating region of the articular surface of the distal humerus. Thus, this area is a good point for screw insertion, especially when using an anterior-to-posterior lag screw; however, caution should be taken in small-statured patients that may have a smaller safe-zone [55].

In a biomechanical study, headless compression screws were demonstrated to provide a more stable fixation of capitellum fractures than four-millimeter partially threaded cancellous lag screws [56]. Both Herbert (Zimmer, Warsaw, Indiana) and Acutrak (Acumed Hillsboro Oregon) screws are options. However, after cyclic testing, which simulated physiologic loading, Acutrak screws inserted in the AP direction perpendicular to the articular surface proved to provide significantly more stable fixation than Herbert screws [57]. This might be due to the fully threaded, tapered shape, and variable pitch of the Acutrak screw. Nevertheless, several authors have reported good clinical outcomes using Herbert screws [58,59,60].

One limitation of the headless screws is the larger diameter due to the cannulation. Watson et al., have proposed the use of locking screws buried within the articular cartilage, as they have a low profile compared to a cortical screw, and very small diameter screws can be used [19].

The ideal orientation of the screws is still debated, and both options have limitations. Placing screws anterior-to-posterior causes damage to the joint surface. Alternatively, screws placed posterior-to-anterior pose a risk to the blood supply of the capitellum and trochlea. In a biomechanical study, the anterior-to-posterior direction provided a more stable fixation [56]. The posterior-to-anterior direction can potentially reduce the risk of collapse of the joint surface along with the subchondral bone, which at times is very thin. With collapse, the physiologic anterior offset of the capitellum is altered with subsequent risk of elbow instability due to the altered relationships between primary and secondary elbow stabilizers [61]. Lopiz et al., reported retrospectively on 18 patients, which were over the age of 65 and treated with either anterior-to-posterior (11 patients) or posterior-to-anterior (nine patients) cannulated screws. At a mean of 4.1 years of follow-up, elbow flexion, Mayo Elbow Performance Index and QuickDASH were slightly better in the posterior-to-anterior group, but the difference was not statistically significant [2]. When anterior-to-posterior orientation is preferred, biodegradable screws might be considered, as they have been shown to provide a similar biomechanical stability as metal screws [62]. However, the risk of reactive synovitis should be considered. Hirvensalo, et al. reported in a case series of eight patients, at a mean of 12 months of follow-up, one patient that developed synovitis that did not affect the final radiographic nor clinical outcomes [63].

#### 4.7.2. Kirschner Wires

Although Kirschner wires have been found to provide lower functional outcomes compared with screws, they are an option, particularly in cases characterized by small fracture fragment size [64,65]. They can be used either isolated or in association with other methods, such as external fixation [66,67]. Threaded Kirschner wires are preferred to smooth ones to minimize the risk of migration. Attention should be paid to avoid joint penetration [9]. Little damage of articular cartilage and easy placement are the main strengths of this device. Disadvantages are possible loosening and the potential need for wire removal. Kirschner wires have been associated with lower arc of motion. This is likely due to the subsequent need of prolonged immobilization after this type of fixation, as they provide only moderately stable fixation [31]. In order to overcome this limitation, the addition of a hinged external fixator has been proposed. He et al., after 42.5 months of follow-up, have retrospectively reported 10 excellent, seven good and three fair results according to the Broberg–Morrey score, with good elbow mobility and no wires loosening [67]. In addition, at 12 months of follow-up, good results have been obtained using fine-threaded Kirschner wires (FFS OrthofixTM, Bussolengo, Italy) due to their autocompression effect, in a cohort of 15 patients reported by Heck et al. [9].

#### 4.7.3. Plates

Posterior comminution has been associated with poorer results, and for patients with these injuries the use of plates, with or without bone grafting, is highly recommended [6,13]. For this fracture type, the rate of nonunion has been reported as high as 33%, especially with associated trochlear involvement [68].

Although Dubberley first suggested the use of pelvic reconstruction plates [6], usually locking plates placed posterolaterally in order to create a sort of new “posterior cortex” through which screws can be inserted, are chosen. This can allow fixation of otherwise unfixable fractures and allow for early range of motion.

In a retrospective case series of 15 patients with Dubberley subtype B fractures treated with posterolateral plate, Wang et al. reported a high rate of fracture healing at a mean follow-up of 32.5 months, with the development of avascular necrosis in one case [14]. The outcome was rated as excellent in 12 patients, good in two, and fair in one. Posterolateral plating can be insufficient in the case of posterior comminution and the augmentation of fixation with bone graft should be considered [6,20,26,35]. Even if proper treatment with the plate is performed, posterior comminution can be associated with worse clinical results, as compared to Dubberley subtype A fractures [69]. A lateral buttress plate can be helpful in cases when comminution is more lateral than posterior [25,66,70]. Moreover, lateral placement of the plate avoids posterior stripping of the humeral surface.

In order to avoid the posterior stripping of the distal humerus, cartilage violation by the screws, and to provide better resistance to the shear force on the coronal plane, the use of an anterior anti-glide plate and posterior-to-anterior or lateral-to-medial screws is an option. A mini-fragment plate or a one-third tubular plate can be contoured to fit anteriorly and its distal portion is bent approximately 60° at the level of the articular surface of the capitellum in a way that it does not interfere with the radial head. The plate is secured using only the most proximal holes, while the distal portion is not fixed and helps neutralize the shear forces across the fracture site [19,25,71]. There is only one clinical study on this technique. Song et al. [71] recently retrospectively reported on their case series of 52 patients, among whom, after a 17.6-month follow-up, 36 were scored excellent, 11 good, and five with fair results, according to the Mayo Elbow Performance Score (MEPS). All fractures were united by a one-year follow-up and three cases sustained hardware loosening or breakage without secondary fracture displacement.

#### 4.7.4. Alternative Treatments

When fracture fragments are too small or thin to be fixed with metal screws, other fixation options should be considered, such as fibrin glue or absorbable pins [53,72,73,74]. Suture fixation is a potential treatment for primarily cartilaginous fractures, such as Bryan–Morrey type 2 [75]. However, the literature on these alternative treatment options only consists of technical notes, case reports, or very small case series.

### 4.8. Elbow Replacement

#### 4.8.1. Total Elbow Arthroplasty (TEA)

To avoid the risk of fixation failure, avascular necrosis, hardware intolerance, non-union, and stiffness, primary TEA is an option for comminuted type 3 and type 4 coronal shear fractures in selected patients [76,77,78]. According to a recently published treatment algorithm on distal humeral fractures [79], TEA is indicated for low demand patients older than 65 with marked osteopenia, severe comminution, and comorbidities.

McKee et al., conducted a study in which they compared ORIF with TEA in forty patients over the age of sixty-five years with comminuted fractures of the distal humerus. They reported better functional outcomes at the two-year follow-up in the group with total elbow arthroplasty based on MEPS and DASH scores, and the results were confirmed at 12.5 years of follow-up [80,81]. However, this treatment should be chosen very carefully, as complications after TEA, ulnar neuropathy, aseptic loosening, triceps failure, and infection can be very challenging to treat. Additionally, the patient can lift no more than 2–3 kg after surgery.

Surgical technique of TEA for shear fractures is similar to that for fractures involving the columns except for the use of the cutting guides, which are not needed in the latter case. TEA can be the best option in cases of secondary failure of ORIF.

There are no reported studies focused specifically on TEA for coronal shear fractures. Some authors have reported on subsets of cases included in larger cohorts of articular distal humeral fractures, and it is difficult to draw conclusions regarding the clinical outcome of TEA for this subtype of injury [82,83,84].

#### 4.8.2. Hemiarthroplasty (HA)

There is continued interest in HA for distal humeral fractures, even though this device is no longer FDA-approved [85,86,87,88,89]. HA is useful in theory for complex shear fractures, as they are characterized by either intact or repairable collateral ligaments. HA could be a viable option for patients that are too young or too active to consider a TEA; however, most results have been reported for elderly patients [90]. We prefer TEA for patients over 65 years of age. Compared to TEA, the HA patient has no weight-lifting restrictions and he or she is not at risk of complications related to the bushing; however, progressive osteoarthritis of the proximal radius and ulna is unique to HA [90]. Although the use of the Kudo and Sorbie humeral components have been reported, the Latitude (Wright Medical, Memphis, TN) is the only implant currently available [91,92]. Clinical studies are limited to small retrospective case series without a specific focus on coronal shear fractures [86,93,94].

#### 4.8.3. Other Prosthetic Options

Lateral elbow resurfacing and capitellar hemi-replacement have been described for treatment of coronal shear fracture sequelae [36,95]. Further studies are needed to understand their application in an acute setting.

## 5. Post-Operative Rehabilitation

The post-operative treatment recommendation varies according to the surgery performed; there are no reports of an evidence-based protocol in the literature to date. Authors have suggested that after fragment excision early range of motion should be encouraged [40,96]. After ORIF, the elbow is mobilized according to bone quality, stability of the fixation, and associated ligamentous injuries [10]. If both fixation and ligaments are stable, early ROM is advised. Active ROM is preferred, as it provides a compressive force to the opposed articular surface and helps maintain joint stability [97,98]. Forearm rotation at 90° of flexion or more is encouraged in the early post-operative period [99]. Although the elbow joint is at high risk of stiffness, a short period of post-operative immobilization is accepted [6,60,100]—a stiff but healed elbow is easier to treat than a fixation failure [10]. Mobilization should start in the over-head position to protect the LUCL. The gravity varus position should be particularly avoided after repair of the LUCL [101,102,103]. Additionally, LUCL protection applies to HA. TEA allows early ROM with weight-lifting restrictions of no more than 4–5 kg as a single event and no more than 1 kg, repeatedly [104].

## 6. Complications

Limited data exists for the long term on surgical excision of the fragments or non-operative management, so most of the complications reported in the literature follow surgical fixation. The rate of complications has been reported to be higher in Dubberley subtype B fractures with posterior comminution than in subtype A [13].

Nonunion is often reported in less than 10% of patient treated surgically [2,6,71]. Nonunion is more common in fractures with involvement of the posterior aspect of the lateral column or trochlea [68] and might be related to the type of hardware used for fixation [66,68]. However, infection is uncommon and usually superficial [35,51,74]. Workup of infection should be performed in cases of nonunion [68].

Symptomatic malunion is very rare, and corrective osteotomy followed by rigid fixation should be considered in the young/middle aged patient—otherwise, total elbow replacement is the best option [105].

**Figure 9:**
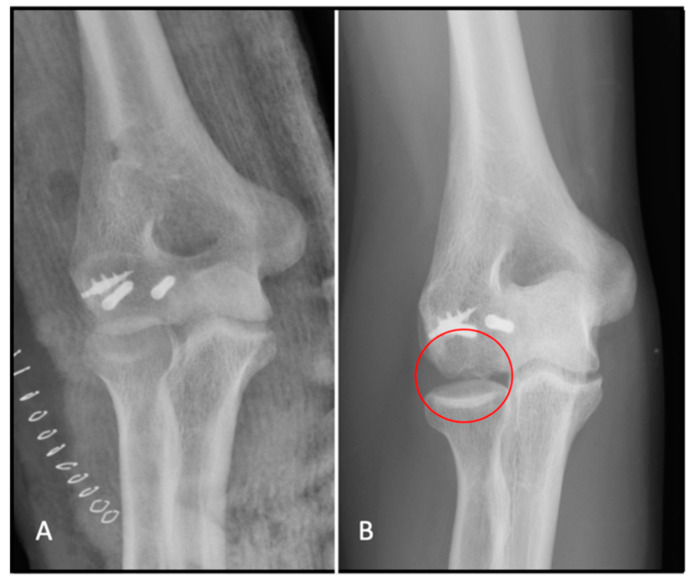
(**A**) Post-operative X-ray; and (**B**) signs of avascular necrosis circled on the follow-up X-ray.

Post-traumatic mild to moderate osteoarthritis occurs between 0% and 32% of cases and can affect both flexion-extension ROM and functional scales [2,6,13,17,26,35,51,66,71]. Arthritis can be subsequent to the acute trauma or iatrogenic, due to fixation through the articular surface, and isolating one from the other is difficult. Avascular necrosis is rare and usually asymptomatic [2,6,13,14,35,51,66] (Figure 9).

Heterotopic ossification is reported in 0% to 38% of cases. They are usually low grade, according to Brooker, and rarely symptomatic [2,6,13,14,26,51,71]. Ulnar neuropathy is rare and can be transient [2].

Elbow instability can be due to either the acute trauma or to surgical exposure, such as releasing the LUCL while extending the Kocher approach [13,61]. Surgical excisions of fracture fragments have limited long-term follow-up reported, although patients may develop progressive valgus instability and chronic pain [37].

Some small case series without complications have been reported [58,59,106]. Although stiffness is common with heterotopic ossification, malunion, prominent implants, infection, arthrosis, and ulnar neuropathy are the attributable causes [48]. In cases where post-traumatic stiffness is present, arthroscopic release might be an option [61]. However, a functional ROM is usually obtained without further surgery.

Table I—list of possible post-operative complications

- Post-traumatic arthritis
- Hardware loosening or breakage
- Nonunion or malunion
- Ulnar neuropathy
- Infections
- Avascular necrosis
- Residual elbow instability
- Stiffness
- Complex regional pain syndrome (CRPS I)

## 7. Conclusions

Coronal shear fractures of the distal humerus are rare but challenging. CT scan is incredibly helpful to plan the treatment, which is typically surgical. The Dubberley classification is the most closely aligned with the determination of treatment. It is hard to draw robust conclusions from the clinical studies available in the literature, as they are mostly case reports or small series. However, fixation with headless compression screws is the most common treatment, with plate and screws being useful in cases with posterior comminution. Elbow replacement is an option for highly comminuted fractures predominantly in the low-demand elderly patient. Complications are not frequent, but a certain degree of stiffness is common.

## Data Availability

Not applicable.
